# Characterization of the GATC regulatory network in *E. coli*

**DOI:** 10.1186/1471-2164-5-48

**Published:** 2004-07-20

**Authors:** Alessandra Riva, Marie-Odile Delorme, Tony Chevalier, Nicolas Guilhot, Corinne Hénaut, Alain Hénaut

**Affiliations:** 1 Laboratoire Génome et Informatique, UMR 8116, CNRS – Université d'Evry Val d'Essonne, Tour Evry 2, 523 Place des Terrasses, 91034 Evry cedex, France; 2 METabolic EXplorer S.A., Biop ô le Clermont-Limagne, 63 360 Saint-Beauzire, France; 3 Previous address: Hong Kong University- Pasteur Research Centre Ltd, Dexter HC Man Building, 8 Sassoon Road, Pokfulam, Hong Kong

## Abstract

**Background:**

The tetranucleotide GATC is methylated in *Escherichia. coli *by the DNA methyltransferase (Dam) and is known to be implicated in numerous cellular processes. Mutants lacking Dam are characterized by a pleiotropic phenotype. The existence of a GATC regulated network, thought to be involved in cold and oxygen shift, had been proposed and its existence has recently been confirmed. The aim of this article is to describe the components of the GATC regulated network of *E. coli *in detail and propose a role of this network in the light of an evolutionary advantage for the organism.

**Results:**

We have classified the genes of the GATC network according to the EcoCyc functional classes. Comparisons with all of *E. coli*'s genes and the genes involved in the SOS and stress response show that the GATC network forms a group apart. The functional classes that characterize the network are the Energy metabolism (in particular respiration), Fatty acid/ Phospholipid metabolism and Nucleotide metabolism.

**Conclusions:**

The network is thought to come into play when the cell undergoes coldshock and is likely to enter stationary phase.

The respiration is almost completely under GATC control and according to our hypothesis it will be blocked at the moment of coldshock; this might give the cell a selective advantage as it increases its chances for survival when entering stationary phase under coldshock. We predict the accumulation of formate and possibly succinate, which might increase the cell's resistance, in this case to antimicrobial agents, when entering stationary phase.

## Background

The tetranucleotide GATC is methylated in *Escherichia. coli *by the DNA methyltransferase (Dam); this enzyme methylates the adenine residue within 5'-GATC-3' sequences in double stranded DNA. GATC motifs and their methylation by Dam play an important role in *E. coli*; they are involved in mismatch repair (see [1] for a review on the subject of mismatch repair and [2] for a review concerning *E. coli *only) and the control of chromosome replication (see [3] for a concise overview on the subject). The methylation state of GATC is also involved in the expression of the *pap *operon; this operon codes for the Pap pili, which are of great importance in the pathogenicity of uropathogenic *E. coli *[4].

**Figure 1:**
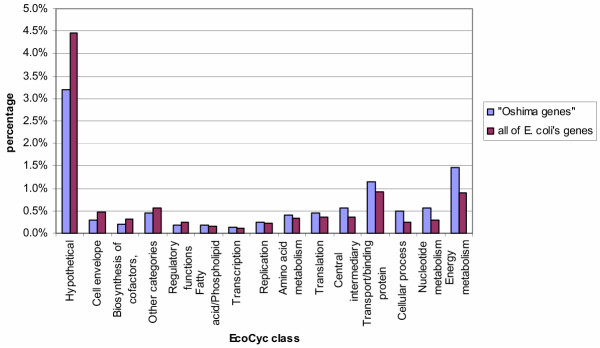
**Comparison of the distribution of the "Oshima genes" with all of *E. coli*'s genes. **The genes of *E. coli *have been classified according to the 15 classes used by EcoCyc. In this figure we compare the distribution of the genes sensitive to the *dam*^+^/ *dam*^- ^background according to a transcriptome analysis carried out by [6] ("Oshima genes") with all of *E. coli*'s genes. The distributions are different (p-value = 1.7 × 10^-7^): the "Oshima genes" contain a much smaller proportion of "Hypothetical" genes, whilst the group "Energy metabolism" is particularly overrepresented.

Mutants that lack Dam are characterized by a pleiotropic phenotype; they show for example an increased sensitivity to DNA-damaging agents, have a higher mutability and increased hyper-recombination [5]. Recent transcriptome analyses on Dam mutants show that nearly 10% of *E. coli*'s genes are affected [6]. When we sort these genes according to their EcoCyc functional classes and compare their distribution with all of *E. coli*'s genes, one can observe that the two distributions are different (p-value = 1.7 × 10^-7^) and that the class Energy metabolism is particularly overrepresented in the genes sensitive to the *dam *genotype (see Figure 1).

In 1996, Hénaut et al. [7] suggested the existence of a GATC regulated network thought to be involved in cold and oxygen shift. Its existence has been recently confirmed by Riva et al. [8], who have worked with a virtual chromosome and used *Salmonella *-a close relative of *E. coli*- as a control organism. The network consists of a number of genes which contain clusters of GATC within their coding sequences.

In fast growing cells (for example in the intestine of their warm blooded host) Dam is a limiting factor and the DNA will be undermethylated [9,10]; this undermethylated DNA possesses an increased melting temperature and thus an increased stability [11].

According to the hypothesis of [7] and [8], the increased stability of the DNA comes into play when the bacteria undergo coldshock (and an oxygen shift), caused by the passage from the intestine to the external environment. At that moment, the transcription of genes containing a GATC cluster will be blocked at the level of the cluster because of the high stability of the hemi-methylated DNA.

The aim of this article is to describe the GATC regulated network of *E. coli *in detail. In order to obtain more information about the characteristics of the network, we compare it with the genes known to be involved in the stress and SOS response. By examining the functions of the genes belonging to the GATC regulated network, we try to address the question about the possible evolutionary advantage of developing a regulatory network controlled by GATC clusters present within the coding regions.

## Results

### Description of the network and its conservation

**Figure 2:**
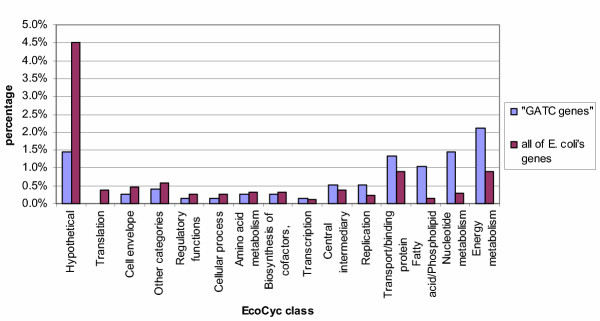
**Comparison of the distribution of the "GATC genes" with all of *E. coli*'s genes. **The genes of *E. coli *have been classified according to the 15 classes used by EcoCyc. In this figure we compare the distribution of the genes containing a GATC cluster ("GATC genes") with all of *E. coli*'s genes. (See Table 2 for the corresponding numerical data.) The distributions are different (p-value = 6 × 10^-15^): the "GATC genes" contain a much smaller proportion of "Hypothetical" genes, whilst the groups "Fatty acid/ Phospholipid metabolism", "Nucleotide metabolism" and "Energy metabolism" are overrepresented.

**Figure 3:**
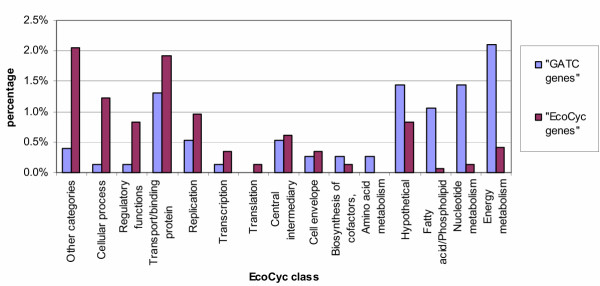
**Comparison of the distribution of the "GATC genes" with the "EcoCyc genes". **The genes of *E. coli *have been classified according to the 15 classes used by EcoCyc. In this figure we compare the distribution of the genes containing a GATC cluster ("GATC genes") with the genes induced under various stress conditions according to EcoCyc ("EcoCyc genes"). (See Table 2 for the corresponding numerical data.) The distributions are different (p-value = 5 × 10^-9^): the "GATC genes" contain a much smaller proportion of genes belonging to "Other categories", genes involved in "Cellular processes" and "Regulatory functions", whilst the groups "Fatty acid/ Phospholipid metabolism", "Nucleotide metabolism" and "Energy metabolism" are overrepresented.

**Figure 4:**
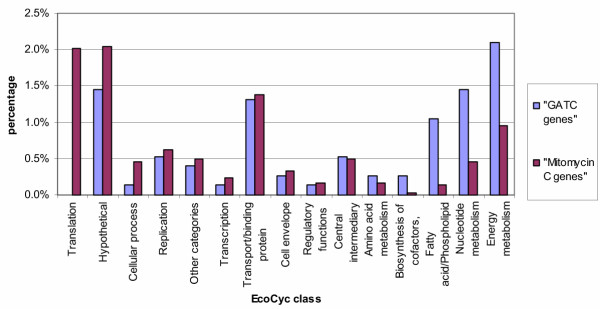
**Comparison of the distribution of the "GATC genes" with the "Mitomycin C genes". **The genes of *E. coli *have been classified according to the 15 classes used by EcoCyc. In this figure we compare the distribution of the genes containing a GATC cluster ("GATC genes") with the genes whose expression is sensitive to the stress caused by the antibiotic mitomycin C (genes involved in the SOS response and other stress response pathways) according to the recent transcriptome analysis carried out by [13] ("Mitomycin C genes"). (See Table 2 for the corresponding numerical data.) The distributions are different (p-value = 2 × 10^-6^): the "GATC genes" contain a much smaller proportion genes involved in "Translation", whilst the groups "Fatty acid/ Phospholipid metabolism", "Nucleotide metabolism" and "Energy metabolism" are overrepresented.

Firstly we assigned each gene in *E. coli *to one of fifteen functional classes according to EcoCyc [12]. This allowed us to examine whether the "GATC genes" (those genes in *E. coli *containing a GATC cluster, listed in Table 1 [see additional file 1]) follow the distribution of all of *E. coli*'s genes or not. The results are displayed in Table 2 and Figure 2 and show that the distribution (in percentage) of the "GATC genes" in the fifteen classes is significantly different from all of *E. coli*'s genes (p-value = 6 × 10^-15^). As the GATC network is thought to come into play when the cell suffers from stress, namely coldshock (coupled to an oxygen shift), we compared the "GATC genes" with genes induced under various stress conditions according to EcoCyc ("EcoCyc genes") as the two groups might overlap. The results of this second comparison are displayed in Table 2 and Figure 3 and show that the distribution (in percentage) of the genes in the fifteen classes is clearly different in the two groups (p-value = 5 × 10^-9^). A third comparison was made, between the "GATC genes" and the group of genes, whose expression is strongly affected by mitomycin C ("Mitomycin C genes") according to a recent transcriptome analysis [13]. Mitomycin C is an antitumor drug and antibiotic which has a strong ability to cause interstrand DNA cross-links [14]. Addition of mitomycin C to a culture of *E. coli *provokes a general stress in the bacteria, affecting almost 30 % of *E. coli*'s genes; 7% of *E. coli*'s genes show particularly strong changes in expression levels, including the genes in the SOS response and genes belonging to other stress response pathways [13]. We have compared this latter group of genes with the "GATC genes". Again the comparison shows the two groups of genes to be distinct (p-value = 2 × 10^-6^, see Table 2, Figure 4).

**Table 2:** Distribution of the different groups of genes according to the EcoCyc functional classification

**Functional class**	**"GATC genes"**	**all of *E. coli*'s genes**	**"EcoCyc genes"**	**"Mitomycin C genes"**
Amino acid metabolism	2	134	0	5
Biosynthesis of cofactors, prosthetic groups, carriers	2	127	2	1
Cell envelope	2	194	5	10
Cellular process	1	102	18	14
Central intermediary metabolism	4	149	9	15
Energy metabolism	16	363	6	29
Fatty acid/Phospholipid metabolism	8	64	1	4
Hypothetical	11	1847	12	62
Nucleotide metabolism	11	120	2	14
Other categories	3	236	30	15
Regulatory functions	1	104	12	5
Replication	4	89	14	19
Transcription	1	47	5	7
Translation	0	150	2	61
Transport/binding protein	10	369	28	42
Total	76	4095	146	303

The table shows the distributions of four groups of genes discussed in this paper, (classified according to the EcoCyc functional classification): the "GATC genes" (genes containing a GATC cluster), all of *E. coli*'s genes, the "EcoCyc genes" (genes induced under various stress conditions according to EcoCyc) and the "Mitomycin C genes" (genes induced by the stress caused by the antibiotic mitomycin C). This table allows the reader to perform the statistical analyses carried out in this paper. The comparison of the distribution of the "GATC genes" with each of the other three groups shows that in all three comparisons three functional classes are overrepresented in the "GATC genes": Energy metabolism, Fatty acid/ Phospholipid metabolism and Nucleotide metabolism. These three classes characterize the GATC regulated network. For an easier interpretation these data are also displayed in the form of histograms in Figures 2, 3 and 4.

The three comparisons show us that the "GATC genes" form a group apart. A characteristic of the GATC network is the overrepresentation of three classes, namely Fatty acid/ Phospholipid metabolism, Nucleotide metabolism and Energy metabolism, indicating that the genes belonging to these three classes are the ones which characterize the network and therefore deserve a closer examination.

Two distinct arguments corroborate the fact that the three classes (Fatty acid/ Phospholipid metabolism, Nucleotide metabolism and Energy metabolism) are at the heart of the GATC Network:

The first argument comes from an analysis of some transcriptome data. Oshima et al. [6] carried out a transcriptome study on *E. coli *in a *dam*^+^/*dam*^- ^background. We have classified the genes affected by this background according to the 15 functional classes in EcoCyc and compared the distribution of these genes with the "GATC genes". At first sight the distribution (in percentage) of the genes in the fifteen classes is different in the two groups (p-value = 1 × 10^-4^). If, however, we do not take into consideration the class "Hypothetical", the two distributions do no longer differ from each other, with a p-value of 0.03 (Table 3 [see additional file 2]. If, on the other hand, we ignore the class "Hypothetical" in the three comparisons mentioned above, i.e. the comparisons of the "GATC genes" with all of *E. coli*'s genes, the stress-induced genes according to EcoCyc and the mitomycin C sensitive genes, the distributions continue to be significantly different.

The second argument stems from the comparison of *E. coli*'s "GATC genes" with the "GATC genes" present in *Salmonella*. *E. coli *contains 76 "GATC genes", *Salmonella *57 (and 3 pseudogenes) and they follow the same distribution in the 15 EcoCyc functional classes (p-value = 0.75).

Twenty-three genes are in common to the two bacteria (i.e. they contain a GATC cluster in both organisms); even if we do not take these genes into account, the rest of the "GATC genes" in the two organisms still follow the same distribution (p-value = 0.33).

We expected the network to be evolutionarily conserved, but not to be identical in the two organisms. A particularly interesting case is given when the same pathway is affected in both organisms, but through different genes, as this represents a particularly strong argument for the evolutionary conservation of the GATC regulated network, beyond the mere conservation of the sequences themselves. The following serve as examples:

The first regards the propionate catabolism, where three genes contain a cluster. *prpE *contains a GATC cluster in both *E. coli *and *Salmonella*. *prpB*, which belongs to the same catabolic pathway, is affected in *E. coli *only. According to our hypothesis, transcription from these two genes would be halted during coldshock. *prpR *codes for the positive regulator of the propionate catabolism operon, to which *prpE *and *prpB *belong to. It contains a cluster in *Salmonella*. According to our hypothesis, transcription of *prpR *would be halted under coldshock, thus inhibiting transcriptional activation of the propionate catabolism operon, effectively blocking propionate catabolism. It is interesting to note that the GATC regulated transcription block acts on two different levels: the genes involved in catabolism, as well as their operon's regulator.

Formate metabolism is affected by GATC clusters in both organisms; according to our hypothesis, the insertion of selenocysteine (required by all three formate dehydrogenases) is blocked in *E. coli *via *selB*, in *Salmonella *through *cysN *(see the corresponding entry in Table 1 [see additional file 1] for details). Furthermore, we find that in *E. coli hyfR *contains a cluster, whose product is required for the induction of the formate hydrogenlyase 2 [15]; by blocking *hyfR*, the formation of the complex would be hindered and thus formate metabolism further inhibited.

In both organisms, a nitrate/ nitrite response regulator is part of the GATC network: *narL *in *E. coli *and *narP *in *Salmonella*. The two enzymes fulfil equivalent roles in respiration, both being involved in the (co-) regulation of a number of genes encoding oxidoreductases and dehydrogenases. The regulated oxidoreductases include a periplasmic nitrite reductase (*nrfABCD*) and two nitrate reductases (*narGHJI *and *napFDAGHBC*); the formate dehydrogenase-N (*fdnGHI*) is regulated by NarL [16-19].

## Discussion

### Evolutionary advantage of GATC network regulation

According to our hypothesis, the GATC network comes into play, when *E. coli *passes from a warm, nutrient rich environment, where it grows rapidly, to a cold, nutrient poor environment, where growth might be expected to be considerably slower, if not completely arrested. This process occurs naturally, when the cells pass from the intestine of their warm blooded host to the external environment. When growing rapidly, the DNA is undermethylated [9,10] and possesses an increased melting temperature and thus an increased stability [11].

We hypothesize that when the bacterium undergoes coldshock, the transcription of genes containing a GATC cluster will be blocked at the level of the cluster because of the high stability of the hemi-methylated DNA.

We will now draw the logical consequences of this hypothesis: if a gene contains a GATC cluster, its transcription will be blocked when the bacterium undergoes coldshock and the product the gene codes for will no longer be formed, affecting the biological process it is involved in.

We will apply this principle systematically to the genes belonging to the three functional classes characterizing the GATC network (Fatty acid/ Phospholipid metabolism, Nucleotide metabolism and Energy metabolism), in order to try answering the following question: is there one and the same selection pressure that can explain why these three particular classes are affected by the GATC network?

#### Nucleotide metabolism

Two main groups of genes can be distinguished: those involved in the synthesis of Nucleotides ("Nucleotide Synthesis", see column "Subclass", Table 1 [see additional file 1]) and those involved in DNA repair ("DNA repair", see column 'Subclass", Table 1 [see additional file 1]). The shut down of macromolecule synthesis such as DNA, RNA and ATP when undergoing coldshock might be seen as the "effort" of the cell to eliminate wasteful energy expenditure, as growth conditions in the new environment will probably be less comfortable than in the host's intestine.

The halt of the DNA repair machinery, on the other hand, might have a different reason: when the cell passes from the intestine to the outside environment, it is halted in the middle of a rapid growth phase, which means that the DNA double helix will be open in certain regions and "loose ends" of newly formed DNA will be present (like, for example, Okazaki fragments). The DNA repair machinery might interpret these fragments as damaged pieces of DNA and proceed to its elimination [20]. An immediate halt of the DNA repair machinery when undergoing coldshock would prevent such "erroneous" repair.

#### Energy metabolism

Again, we will look at two classes of genes, which are particularly interesting: those involved in respiration and those involved in the metabolism of succinate ("Respiration" and "Succinate", column "Subclass", Table 1 [see additional file 1]).

##### Respiration

**Figure 5:**
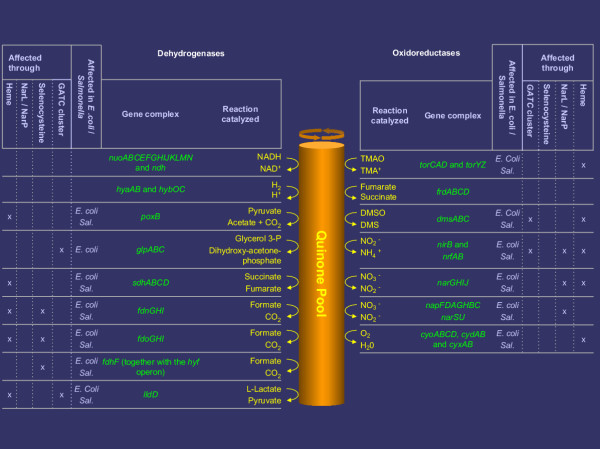
**The respiratory components of *E. coli *and *Salmonella *. **This figure gives an overview of the respiration of *E. coli *and *Salmonella *and how it is influenced by the GATC network. As can be seen, the large majority of enzymes involved in respiration are affected, directly or indirectly, by the GATC network. Listed on the left hand side are the gene complexes that make up the dehydrogenases, together with the reactions they catalyse. The resulting reducing equivalents are passed through the common quinone pool and are used by the oxidoreductases, listed on the right hand side, together with the reactions they catalyse. Also given are the means by which the various dehydrogenases and oxidoreductases are affected through the GATC cluster network. "Heme": the gene complex contains a heme, whose synthesis is blocked by a GATC cluster in *gltX*. "NarL/ NarP" the gene complex is under control by NarL/ NarP whose respective genes are blocked by a GATC cluster. "Selenocysteine": the gene complex contains selenocysteine whose insertion into the protein is blocked (GATC clusters in *selB*/ *cysN*). "GATC cluster": the gene complex itself contains a GATC cluster. For further details please refer to the results section."*Sal*.": *Salmonella*.

The respiratory system of *E. coli *has a modular character (see [21] for a comprehensive introduction to the subject). There are three types of respiratory components (see Figure 5):

1) Substrate specific dehydrogenases which oxidize their substrates and feed electrons to the mobile quinone pool.

2) Quinones, which deliver reducing equivalents to the terminal oxidoreductases.

3) Terminal oxidoreductases which reduce the terminal electron acceptors.

The heme synthesis is blocked in *E. coli *(and *Salmonella*) at the level of the aminolevulinic acid (ALA) production. ALA is synthesized from glutamate through the action of GltX, whose corresponding gene, *gltX*, contains a GATC cluster (in both organism). Hemes play a fundamental role in the energy conserving electron transport chains and are also present as cofactors in a number of enzymes [22]. Thus, if heme synthesis is blocked, the repercussions are felt by the heme containing enzymes, notably by the **dehydrogenases ***poxB*, *sdhABCD*, *fdnGHI*, *fdoGHI*, *fdhF *and *lldD *and the **oxidoreductases ***torCAD*, *torYz*, *nirB*, *nrfAB*, *cyoABCD*, *cydAB *and *cyxAB.*

Respiration is further affected through *narL*, containing a GATC cluster in *E. coli *(in *Salmonella *it is *narP *which contains a GATC cluster) which codes for a **nitrate / nitrite response regulator **and is involved in the (co-) regulation of a number of genes encoding oxidoreductases and dehydrogenases. The oxidoreductases regulated include a periplasmic nitrite reductase (*nrfABCD*) and two nitrate reductases (*narGHJI *and *napFDAGHBC*); the formate dehydrogenase-N (*fdnGHI*) is regulated by NarL [15-18].

As discussed in the section above, the formate dehydrogenases require selenocysteine, whose incorporation is halted. We can thus expect formate to accumulate in the cell when it undergoes coldshock, an aspect discussed further below.

Two more dehydrogenases – *glpA *and *lldD *– and oxidoreductases – *dmsA *and *nirB*- are blocked in *E. coli*.

If we now look at the respiration as a whole, at all the respiratory enzymes affected by the clusters, directly or indirectly, the following picture emerges (see Figure 5): the great majority of enzymes are blocked.

Interestingly, Nyström [23] points out that during aerobic starving conditions or stasis the aerobic respiration in *E. coli *is blocked (by ArcA). Applying our hypothesis we come to the conclusion that genes involved in aerobic respiration are blocked and moreover, that almost the entire respiration, aerobic as well as anaerobic, is brought to a standstill.

A possible explanation for this can be found in the work of Dukan & Nyström [24]. They point to the fact that when encountering stress (oxidative, nutritive, osmotic or thermal) the cell is likely to enter the stationary phase. Dukan & Nyström studied *E. coli *cultures when entering stationary phase under different conditions; they found that cell viability depends on the conditions the cell was prior to entering the stationary phase. Cells entering the stationary phase aerobically showed a very high mortality. Cells entering the stationary phase anaerobically, though, showed a much higher viability. Seen from this perspective it could be that what we predict to happen through the GATC regulation is the "attempt" of the cell to stay in an "anaerobic mode": the bacterium does not, for example, start producing enzyme complexes needed for aerobic respiration. Blocking respiration (when entering stationary phase) might be a preventive measure because all respiration, in the presence of oxygen, is a potential danger as it can lead to the production of free radicals and peroxide.

##### Metabolism of succinate

In the section above, we predict that formate probably accumulates in the cell when undergoing coldshock. A second prediction is that succinate might also be accumulating. If we look at the TCA cycle in anaerobiosis we note that the reductive branch (leading from oxaloacetate to succinyl CoA) is not affected by GATC clusters except for the last step, leading from succinate to succinyl CoA. Thus, we expect succinate to accumulate during coldshock. (In *Salmonella*, a recently discovered pathway leading from succinate to propionate is also blocked through *ygfH *(see the relevant entry in Table 1 [see additional file 1]), also suggesting an accumulation of succinate in the cell.) As mentioned above, Dukan & Nyström point out that a cell undergoing stress (including coldshock) is likely to enter stationary phase; this coupled with recent work carried out on *Salmonella *and *E. coli *[25] might give an explanation: the authors suggest that when the cells are in stationary phase, an accumulation of formate and succinate has a protective effect against antimicrobial agents. The mechanisms and exact nature of this protection are not yet understood.

##### Fatty acid / Phospholipid metabolism

We can make two observations regarding this group. The first is, that the synthesis of two enzymes which are essential for fatty acid synthesis are under GATC cluster control: the biosynthesis of ACP, the central coenzyme of fatty acid biosynthesis [26] is halted through *acpS *and *ilvD *(see the relevant entry in Table 1 [see additional file 1]); furthermore, the synthesis of biotin, an essential cofactor in fatty acid synthesis, is hindered through *mioC*, which contains a GATC cluster (recent work suggesting that MioC is an essential cofactor for the biotin synthase [27]). The second observation regards the metabolism of propionate. As mentioned above, propionate catabolism is halted in both *E. coli *and *Salmonella*. Propionate, a short chain fatty acid (SCFA) is present in relative abundance in the warm blooded hosts' intestine [28,29], and less so in the external environment. It might be, that the GATC network acts to quickly stop a catabolic process for which it will have less use once in the external environment.

### The (control) mechanism

So far we have stressed the importance of the physical property of hemi- or unmethylated GATC motifs, especially when present in clusters: the increased stability of the DNA, which comes into play when the ambient temperature of the bacterium is suddenly lowered.

We hypothesize that this increased stability hinders transcription of the gene containing a GATC cluster when the cell undergoes coldshock and is likely to enter stationary phase; we have looked at the effect this might have on the cell.

A question we might ask is that of the control mechanism: apart from the control exerted by the physical property of hemi- or unmethylated GATC motifs, is there something else that recognizes these motifs? The protein SeqA, for example, recognizes hemimethylated GATC sequences placed on the same face of the DNA double helix and it does so in a cooperative, histone-like manner, forming a homotetramer [30]. It might indeed be that the GATC cluster network also requires the intervention of such a protein in order to fully exert its functions.

This question, however, reaches beyond the abilities of the research *in silico *and would require an intervention from the wet lab.

## Conclusions

*E. coli *and *Salmonella *possess a GATC cluster regulated network. The clusters are found within the coding sequences and their distribution is not at random. Three functional classes characterize the network: Nucleotide metabolism, Energy metabolism and Fatty acid / Phospholipid metabolism.

We hypothesize that the network comes into play when the cell passes from the warm, nutrient rich environment of its warm blooded host's intestine to the external environment, i.e., when the cell undergoes coldshock and is likely to enter stationary phase.

According to our theory, the transcription of genes containing a GATC cluster will be blocked at the level of the cluster when the bacterium undergoes coldshock and the product the gene codes for will no longer be formed, affecting the biological process it is involved in.

We have applied this principle to the three functional classes that characterize the GATC network and come to the conclusion that respiration is almost completely under GATC control and will be blocked at the moment of coldshock; this might give the cell a selective advantage as it increases its chances for survival when entering stationary phase under coldshock. We also predict the accumulation of formate and possibly succinate, which might increase the cell's resistance, in this case to antimicrobial agents, when entering stationary phase.

## Methods

### Procedure

The procedure has been described elsewhere [8], here a general outline:

We have taken a classic genomic approach, analyzing the statistical distribution of GATC along the chromosome, using a realistic model of the chromosome as theoretical reference. We thus identify local enrichments, or clusters, in the real chromosome. A GATC cluster is identified in the following manner: GATC pairs separated by less than 8 bp and triplets in regions spanning less than 62 bp are kept in a preliminary screening. In a second step we retain only those regions where there are at least four GATC motifs and where the average distance between pairs is shorter than 18 bp.

We confirm the presence of GATC clusters within the genes. In order to verify that the particular distribution observed in *E. coli *is not a statistical artefact, but has a physiological role, we have carried out the same analysis on *Salmonella*, making the hypothesis that the genes containing a GATC clusters should be largely the same in the two bacteria. This has been indeed observed, showing that the genes containing a GATC cluster are part of a regulation network

We thus obtain a list of genes for *E. coli *(76) and for *Salmonella *(57 and three pseudo genes) containing GATC clusters, displayed in Table 1 [see additional file 1]. With the help of the data mining tools listed below, we try to attribute a function to each of these genes and group them into classes according to EcoCyc's system [12]. For certain genes, which have more than one function, we have chosen the class based on the particular context of the current study (see Table 1 [see additional file 1] for details).

In order to compare the genes belonging to *E. coli*'s GATC network (the "GATC genes", Table 4 [see additional file 3], column F) with those involved in the stress and SOS response, we retrieved the genes listed under "adaptation" and "SOS response" at EcoCyc (the "EcoCyc genes", Table 4 [see additional file 3], column D). We also retrieved he genes whose expression is sensitive to mitomycin C, which in addition to the SOS response also includes genes belonging to other stress response pathways, according to a recent transcriptome analysis carried out by [12] (the "Mitomycin C genes", Table 4 [see additional file 3], column E). Again, the "EcoCyc genes" and "Mitomycin C genes" were classified according to EcoCyc's system. A summary of the results is displayed in Table 2.

### Data mining tools

In order to gain information about the "GATC genes" identified, we have used the following databases:

• EcoCyc [12],

• KEGG [34],

• METAVISTA^® ^[35](proprietary data base of the Metabolic Explorer society),

• PubMed [36],

• Swiss-Prot [37] using the SRS search tool to interrogate the SWALL (SPTR) database (accessible as SWall on the SRS server).

## Authors' contributions

AR performed the molecular genetic studies, participated in the statistical analysis and drafted the manuscript, MOD participated in the design of the study and the statistical analysis, TC, NG and CH were responsible for the data mining, AH conceived the study, participated in its design and coordination. All authors read and approved the final manuscript.

## Supplementary Material

Additional File 1 **The "GATC genes" in *E. coli *and *Salmonella ***The table lists all genes that contain a GATC cluster in *E. coli *or in *Salmonella*. **ORF(#ID): **the ORF number and #ID of the genes according to Oshima et al. [6]. Empty cells indicate genes not analyzed in their work. The column "**EcoCyc genes**" denotes the genes induced under various stress conditions according to EcoCyc, "**Mitomycin C genes**" denotes the genes induced by the stress caused by the antibiotic mitomycin C (genes involved in the SOS response and other stress response pathways) according to the recent transcriptome analysis carried out by [13], the two columns "**GATC genes**" denote genes containing a GATC cluster (in *E. coli *or in *Salmonella*). The column "**Oshima genes**" denotes genes in *E *coli sensitive to the *dam*^+^/ *dam*^- ^background. **"EcoCyc functional class (modified)" **gives the genes, classified according to the EcoCyc functional classes, with the modifications made by us (changes are justified in the last column). "**Subclass**" refers to the various groups of genes discussed in the results/ discussion section of this paper. **"1"**: the gene is affected, **"0"**: the gene is not affected, **"N.A."**: no information was available. Click here for file

Additional File 2 **Distribution of the different groups of genes according to the EcoCyc functional classification, without the class "Hypothetical" **The table shows the distributions of the five groups of genes discussed in this paper, (classified according to the EcoCyc functional classification) after removal of the class "Hypothetical". The five groups are: the "GATC genes" (genes containing a GATC cluster), all of *E. coli*'s genes, the "EcoCyc genes" (genes induced under various stress conditions according to EcoCyc), the "Mitomycin C genes" (genes induced by the stress caused by the antibiotic mitomycin C) and the "Oshima genes" (genes sensitive to the *dam*^+^/ *dam*^- ^background). The distributions of the "GATC genes" and the "Oshima genes" do not differ from each other (p-value = 0.03). The comparisons of the "GATC genes" with all of *E. coli*'s, genes, the "EcoCyc genes" and the "Mitomycin C genes", however, show that the distributions continue to be significantly different, even after the removal of the class "Hypothetical". Click here for file

Additional File 3 The file contains the raw data used for this article and allows the reader to re-trace all the calculations made Click here for file
