# Supplementary material for: Characterization of the GATC regulatory network in E. coli
Source: BMC Genomics. 2004 Jul 20;5:48. doi: 10.1186/1471-2164-5-48 (PMC493266; doi:10.1186/1471-2164-5-48)
Supplement: Additional File 1 — The "GATC genes" in E. coli and Salmonella The table lists all genes that contain a GATC cluster in E. coli or in Salmonella. ORF(#ID): the ORF number and #ID of the genes according to Oshima et al. [6]. Empty cells indicate genes not analyzed in their work. The column "EcoCyc genes" denotes the genes induced under various stress conditions according to EcoCyc, "Mitomycin C genes" denotes the genes induced by the stress caused by the antibiotic mitomycin C (genes involved in the SOS response and other stress response pathways) according to the recent transcriptome analysis carried out by [13], the two columns "GATC genes" denote genes containing a GATC cluster (in E. coli or in Salmonella). The column "Oshima genes" denotes genes in E coli sensitive to the dam+/ dam- background. "EcoCyc functional class (modified)" gives the genes, classified according to the EcoCyc functional classes, with the modifications made by us (changes are justified in the last column). "Subclass" refers to the various groups of genes discussed in the results/ discussion section of this paper. "1": the gene is affected, "0": the gene is not affected, "N.A.": no information was available. [file 1471-2164-5-48-S1.pdf]

ORF(#ID)	cds name of *E. coli* or *Salmonella*	SPTr_ID for *E. coli*	SPTr_ID for *Salmonella*	"EcoCyc genes" *E. coli*	"Mitomycin C genes" *E. coli*	"GATC genes" *E. coli*	"GATC genes" *Salmonella*	"Oshima genes" *E. coli*	EcoCyc functional class	EcoCyc functional class (modified)	Subclass (where applicable)	justification for change of functional class (where applicable)
JW2326 (409#1)	*aroC*	AROC_ECOLI		0	0	1	0	0	Amino acid metabolism	Amino acid metabolism		
JW1734 (327#8)	*astB*		Q8Z6G2	0	0	0	1	1	Amino acid metabolism	Amino acid metabolism		
JW3911 (537#1)	*metL*	AK2H_ECOLI		0	0	1	0	0	Amino acid metabolism	Amino acid metabolism		
JW0436 (150#3)	*cof*	COF_ECOLI	Q8Z8U7	0	0	1	1	0	Hypothetical	Biosynthesis of cofacters, prosthetic groups, carriers		The relevant SWALL entry gives: "Possible role in ferric uptake"
JW0586 (165#9)	*entE*	ENTE_ECOLI	Q8Z8L1	0	0	1	1	1	Biosynthesis of cofacters, prosthetic groups, carriers	Biosynthesis of cofacters, prosthetic groups, carriers		
JW0372 (142#7)	*ddlA*	DDLA_ECOLI	DDLA_SALTY	0	0	1	1	0	Cell envelope	Cell envelope		
JW4192 (657#19)	*mpl*		Q8Z145	0	0	0	1	0	Cell envelope	Cell envelope		
JW2503 (429#3.1)	*pbpC*	PBPC_ECOLI		0	0	1	0	0	Cell envelope	Cell envelope		
JW3914 (537#3)	*katG*	CATA_ECOLI		0	1	1	0	0	Celluler process	Cellular process		
JW1878 (339#5)	*motB*		Q8Z5U7	0	0	0	1	1	Celluler process	Cellular process		
JW4200 (658#1)	*treR*		Q8Z133	1	0	0	1	0	Celluler process	Cellular process		
JW0181 (123#1)	*ldcC*	DCLZ_ECOLI	Q8Z998	0	0	1	1	0	Central intermediary metabolism	Central intermediary metabolism		
JW3195 (526#1)	*nanR*	NANR_ECOLI		0	0	1	0	0	Central intermediary metabolism	Central intermediary metabolism		
JW4053 (643#4)	*phnP*	PHNP_ECOLI		0	0	1	0	0	Central intermediary metabolism	Central intermediary metabolism		
JW4185 (657#12)	*ppa*	IPYR_ECOLI		0	0	1	0	0	Central intermediary metabolism	Central intermediary metabolism		

JW2721 (452#7)	*cysN*		CYSN_SALTI	0	0	0	1	0	Central intermediary metabolism	Energy metabolism	Respiration	The enzyme is implicated in the cysteine biosynthetic pathway. However, its counterpart in E. coli, CysN, is also required for the formation of selenocysteine tRNA (KEGG) and thus required for the formate dehydrogenases.
JW0877 (215#7)	*dmsA*	DMSA_ECOLI	Q8Z812	0	0	1	1	1	Energy metabolism	Energy metabolism	Respiration	
JW2235 (378#7)	*glpA*	GLPA_ECOLI		0	0	1	0	0	Energy metabolism	Energy metabolism	Respiration	
JW2395 (417#3)	*gltX*	SYE_ECOLI	SYE_SALTI	0	0	1	1	0	Translation	Energy metabolism	Respiration	GltX is required for ALA (aminolevulinic acid) synthesis, which is fundamental for heme synthesis and thus important for a number of respiratory components.
JW2476 (425#4)	*hyfR*	HYFR_ECOLI		0	0	1	0	0	Translation	Energy metabolism	Respiration	[15]
JW3580 (575#3)	*lldD*	LLDD_ECOLI		0	0	1	0	1	Energy metabolism	Energy metabolism	Respiration	
JW1212 (248#11)	*narL*	NARL_ECOLI		0	0	1	0	0	Energy metabolism	Energy metabolism	Respiration	
JW2181 (372#1)	*narP*		Q8Z576	0	0	0	1	0	Energy metabolism	Energy metabolism	Respiration	
JW3328 (624#7)	*nirB*	NIRB_ECOLI	Q8Z1Z9	0	0	1	1	1	Energy metabolism	Energy metabolism	Respiration	
JW3563 (577#4)	*selB*	SELB_ECOLI	Q8Z2D7	0	0	1	1	0	Translation	Energy metabolism	Respiration	SWALL: "Translation factor necessary for the incorporation of selenocysteine into proteins". These proteins are the formate dehydrogenases (involved in respiration).
JW3934 (535#8)	*sthA (udhA)*	STHA_ECOLI	STHA_SALTY	0	0	1	1	1	Energy metabolism	Energy metabolism	Respiration	
JW3975 (631#1)	*aceA; icl*		Q8Z1V9	0	0	0	1	1	Central intermediary metabolism	Energy metabolism	Succinate	Part of the TCA cycle and therefore placed in this group (like sucD).
JW0718 (177#3)	*sucD*	SUCD_ECOLI	Q8Z8C5	0	1	1	1	1	Energy metabolism	Energy metabolism	Succinate	
JW2887 (471#10)	*ygfH*	YGFH_ECOLI		0	0	1	0	0	Hypothetical	Energy metabolism	Succinate	[31]
JW1228 (251#7)	*adhE*	ADHE_ECOLI		0	1	1	0	1	Energy metabolism	Energy metabolism		
JW2894 (473#1)	*epd; gapB*		Q8XGA4	0	0	0	1	0	Energy metabolism	Energy metabolism		
JW0740 (179#8)	*galK*	GAL1_ECOLI		1	0	1	0	0	Energy metabolism	Energy metabolism		
JW3391 (616#2)	*glgP*	PHSG_ECOLI		0	0	1	0	0	Energy metabolism	Energy metabolism		
	maeB	MAO2_ECOLI		0	0	1	0	N.A.	N.A.	Energy metabolism		

JW3062 (512#10)	*uxaA*	UXAA_ECOLI		0	0	1	0	0	Energy metabolism	Energy metabolism		
JW2547 (434#6)	*acpS*	ACPS_ECOLI		0	0	1	0	0	Fatty acid/Phospholipid metabolism	Fatty acid/Phospholipid metabolism	Coenzyme/cofactor	
JW3744 (556#5)	*ilvD*	ILVD_ECOLI		0	0	1	0	0	Amino acid metabolism	Fatty acid/Phospholipid metabolism	Coenzyme/cofactor	The enzyme has two functions: the first in the amino acid metabolism (valine and isoleucine), the second in the biosynthesis of ACP. We emphasize this second function.
JW3720 (560#16)	*mioC*	MIOC_ECOLI		0	1	1	0	0	Replication	Fatty acid/Phospholipid metabolism	Coenzyme/cofactor	[27]
JW2704 (450#5)	*prpB*	PRPB_ECOLI		1	0	1	0	0	Hypothetical	Fatty acid/Phospholipid metabolism	Propionate	part of the same catabolic pathway as prpE.
JW0326 (138#1)	*prpE*	PRPE_ECOLI	Q8Z902	0	0	1	1	0	Fatty acid/Phospholipid metabolism	Fatty acid/Phospholipid metabolism	Propionate	
JW0322 (137#7)	*prpR*		Q8Z906	0	0	0	1	0	Regulatory functions	Fatty acid/Phospholipid metabolism	Propionate	the transcriptional regulator of the prp operon, hence the placement.
JW2433 (421#5)	*eutC*	EUTC_ECOLI		0	0	1	0	0	Fatty acid/Phospholipid metabolism	Fatty acid/Phospholipid metabolism		
JW0408 (146#11)	*pgpA*	PGPA_ECOLI		0	0	1	0	0	Fatty acid/Phospholipid metabolism	Fatty acid/Phospholipid metabolism		
JW0482 (155#2)	*ybbO*	YBBO_ECOLI	Q8XGS4	0	0	1	1	1	Fatty acid/Phospholipid metabolism	Fatty acid/Phospholipid metabolism		
JW0080 (109#10)	*mraW*		MRAW_SALTI	0	0	0	1	0	Hypothetical	Hypothetical		
JW2635 (443#4)	*STY2910*		Q8Z4F9	0	0	0	1	1	Hypothetical	Hypothetical		
JW3819 (550#14)	*STY3047*		Q8Z475	0	0	0	1	0	Hypothetical	Hypothetical		
JW2731 (453#3)	*STY3071*		Q8Z461	0	0	0	1	0	Hypothetical	Hypothetical		
JW2983 (505#13)	*STY3342*		Q8Z3P9	0	0	0	1	0	Hypothetical	Hypothetical		
JW3453 (609#1)	*STY4224*		Q8Z260	0	0	0	1	0	Hypothetical	Hypothetical		

	ybgG	YBGG_ECOLI		0	0	1	0	N.A.	N.A.	Hypothetical			
JW2166 (370#7)	*yejB*	YEJB_ECOLI		0	0	1	0	0	Hypothetical	Hypothetical			
JW2227 (377#1)	*yfaL*	YFAL_ECOLI		0	0	1	0	0	Hypothetical	Hypothetical			
	yfhM	YFHM_ECOLI		0	0	1	0	N.A.	N.A.	Hypothetical			
	yghJ	ACFD_ECOLI		0	0	1	0	N.A.	N.A.	Hypothetical			
JW3006 (507#9)	*ygiC*		Q8XFP6	0	0	0	1	0	Hypothetical	Hypothetical			
JW3853 (544#5)	*yihU*	YIHU_ECOLI	Q8Z2T6	0	0	1	1	0	Hypothetical	Hypothetical			
JW3989 (633#9)	*yjbH*	YJBH_ECOLI	Q8Z1U4	0	0	1	1	0	Hypothetical	Hypothetical			
JW4190 (657#17)	*yjfF*	YJFF_ECOLI		0	0	1	0	0	Hypothetical	Hypothetical			
JW4138 (654#1)	*yjfH*	YJFH_ECOLI	Q8Z182	0	0	1	1	0	Hypothetical	Hypothetical			
JW4299 (668#8)	*yjiN*	YJIN_ECOLI		0	0	1	0	0	Hypothetical	Hypothetical			
JW0658 (171#5.1)	*yleA*	YLEA_ECOLI		0	0	1	0	0	Hypothetical	Hypothetical			
	mod		Q8Z913	N.A.	N.A.	0	1	N.A.	N.A.	Hypothetical			
	STY0042		Q8Z9N9	N.A.	N.A.	0	1	N.A.	N.A.	Hypothetical			
	STY0099		Q8Z9K2	N.A.	N.A.	0	1	N.A.	N.A.	Hypothetical			
	STY0319		Q8Z954	N.A.	N.A.	0	1	N.A.	N.A.	Hypothetical			
	STY3047		Q8Z475	N.A.	N.A.	0	1	N.A.	N.A.	Hypothetical			
	STY3536		Q8Z3E3	N.A.	N.A.	0	1	N.A.	N.A.	Hypothetical			
	STY4415		Q8Z1U9	N.A.	N.A.	0	1	N.A.	N.A.	Hypothetical			
	STY4459		Q8Z1S1	N.A.	N.A.	0	1	N.A.	N.A.	Hypothetical			
JW3962 (532#1)	*nfi*	NFI_ECOLI		0	0	1	0	0	Transcription	Nucleotide metabolism	DNA repair	SWALL: "Selectively cleaves single-stranded DNA or duplex DNA containing uracil or that is damaged by a variety of agents….could act in DNA repair".	
JW2788 (460#1)	*recB*	EX5B_ECOLI		0	0	1	0	0	Nucleotide metabolism	Nucleotide metabolism	DNA repair		
JW2787 (459#9)	*recD*	EX5A_ECOLI		0	0	1	0	0	Nucleotide metabolism	Nucleotide metabolism	DNA repair		
JW4019 (637#7)	*uvrA*	UVRA_ECOLI		1	1	1	0	1	Replication	Nucleotide metabolism	DNA repair	SWALL: "the ABC excision nuclease is a DNA repair enzyme that catalyzes the excision reaction of UV-damaged nucleotide segments".	
JW2008 (351#4)	*hisI (hisIE)*	HIS2_ECOLI	HIS2_SALTI	0	0	1	1	0	Amino acid metabolism	Nucleotide metabolism	Nucleotide synthesis	Involved in two steps of the histidine biosynthetic pathway which are in common with the purine biosynthetic pathway.	
JW3969 (532#8)	*purD*		Q8Z334	0	0	0	1	1	Nucleotide metabolism	Nucleotide metabolism	Nucleotide synthesis		
JW2309 (406#15)	*purF*	PUR1_ECOLI	Q8Z503	0	0	1	1	1	Nucleotide metabolism	Nucleotide metabolism	Nucleotide synthesis		

JW0928 (221#13)	*pyrD*	PYRD_ECOLI		0	0	1	0	0	Nucleotide metabolism	Nucleotide metabolism	Nucleotide synthesis	
JW4203 (658#4)	*pyrI*	PYRI_ECOLI		0	0	1	0	0	Nucleotide metabolism	Nucleotide metabolism	Nucleotide synthesis	
JW4313 (670#8)	*hsdR; hsr*	T1RK_ECOLI	Q8Z0W6	0	1	1	1	0	Nucleotide metabolism	Nucleotide metabolism		
JW2553 (435#3)	*lepA*	LEPA_ECOLI	LEPA_SALTY	0	0	1	1	0	Nucleotide metabolism	Nucleotide metabolism		
JW1793 (333#10)	*rnd*	RND_ECOLI		0	0	1	0	0	Nucleotide metabolism	Nucleotide metabolism		
JW0557 (162#3)	*nfrA*	NFRA_ECOLI		0	0	1	0	0	Hypothetical	Other categories		as it has been assigned a SPTrembl ID, "Other categories" seems more justified.
JW2507 (429#8)	*pepB*	PEPB_ECOLI		0	0	1	0	0	Other categories	Other categories		
	psiE	PSIE_ECOLI		0	0	1	0	N.A.	N.A.	Other categories		
JW3000 (507#3)	*STY3361*		Q8Z3P0	0	0	0	1	0	Other categories	Other categories		
JW2064 (358#2)	*baeR*	BAER_ECOLI		0	0	1	0	0	Regulatory functions	Regulatory functions		
JW2212 (374#3)	*rcsC*		RCSC_SALTI	0	0	0	1	0	Regulatory functions	Regulatory functions		
JW3212 (528#1)	*yhcS*		Q8XFH1	0	0	0	1	0	Regulatory functions	Regulatory functions		
JW3679 (565#10)	*dnaA*	DNAA_ECOLI	DNAA_SALTI	0	0	1	1	0	Replication	Replication		
JW4325 (672#5)	*dnaC*	DNAC_ECOLI	Q8Z0W1	0	0	1	1	0	Replication	Replication		
JW0905 (218#8)	*mukF*	MUKF_ECOLI		0	0	1	0	0	Celluler process	Replication		[32,33]
JW2998 (507#1)	*parE (nfxD)*	PARE_ECOLI	PARE_SALTY	0	0	1	1	0	Replication	Replication		
JW2481 (426#5)	*yfgE*		Q8XEQ0	0	0	0	1	0	Replication	Replication		
JW0058 (106#11)	*hepA*	HEPA_ECOLI		0	0	1	0	0	Transcription	Transcription		
JW3133 (519#2)	*pnp*		Q8Z3I0	0	0	0	1	0	Transcription	Transcription		
JW0836 (210#10)	*rimK*		RIMK_SALTY	0	1	0	1	0	Translation	Translation		
JW0396 (145#4)	*tgt*		TGT_SALTI	0	0	0	1	0	Translation	Translation		
JW3106 (517#2)	*agaY*	AGAY_ECOLI		0	0	1	0	0	Transport/binding protein	Transport/binding protein		
JW4166 (655#15)	*cycA; dagA*		Q8Z160	0	0	0	1	0	Transport/binding protein	Transport/binding protein		
JW3512 (603#13)	*dppB*	DPPB_ECOLI		0	0	1	0	1	Transport/binding protein	Transport/binding protein		

JW0103 (112#3)	*hofB*	HOFB_ECOLI		0	0	1	0	0	Transport/binding protein	Transport/binding protein		
JW1613 (315#3)	*malX*	PTOA_ECOLI		0	0	1	0	0	Transport/binding protein	Transport/binding protein		
JW4201 (658#2)	*mgtA*	ATMA_ECOLI		0	0	1	0	0	Transport/binding protein	Transport/binding protein		
JW3226 (529#7)	*panF*	PANF_ECOLI	Q8Z3D2	0	0	1	1	0	Transport/binding protein	Transport/binding protein		
JW4066 (644#1)	*phnD*	PHND_ECOLI		0	0	1	0	0	Transport/binding protein	Transport/binding protein		
JW2797 (461#7)	*ptsP*		Q8Z411	0	0	0	1	0	Transport/binding protein	Transport/binding protein		
JW3315 (625#6)	*yheS*	YHES_ECOLI		0	0	1	0	0	Transport/binding protein	Transport/binding protein		
	ypdD	YPDD_ECOLI		0	0	1	0	N.A.	N.A.	Transport/binding protein		
	zitB	ZITB_ECOLI		0	0	1	0	N.A.	N.A.	Transport/binding protein		
