# Supplementary material for: Characterization of the GATC regulatory network in E. coli
Source: BMC Genomics. 2004 Jul 20;5:48. doi: 10.1186/1471-2164-5-48 (PMC493266; doi:10.1186/1471-2164-5-48)
Supplement: Additional File 2 — Distribution of the different groups of genes according to the EcoCyc functional classification, without the class "Hypothetical" The table shows the distributions of the five groups of genes discussed in this paper, (classified according to the EcoCyc functional classification) after removal of the class "Hypothetical". The five groups are: the "GATC genes" (genes containing a GATC cluster), all of E. coli's genes, the "EcoCyc genes" (genes induced under various stress conditions according to EcoCyc), the "Mitomycin C genes" (genes induced by the stress caused by the antibiotic mitomycin C) and the "Oshima genes" (genes sensitive to the dam+/ dam- background). The distributions of the "GATC genes" and the "Oshima genes" do not differ from each other (p-value = 0.03). The comparisons of the "GATC genes" with all of E. coli's, genes, the "EcoCyc genes" and the "Mitomycin C genes", however, show that the distributions continue to be significantly different, even after the removal of the class "Hypothetical". [file 1471-2164-5-48-S2.pdf]

**Functional class**	**"GATC genes"**	**all of *E. coli*'s genes**	**"EcoCyc genes"**	**"Mitomycin C genes"**	**"Oshima genes"**
Amino acid metabolism	2	134	0	5	14
Biosynthesis of cofactors, prosthetic groups, carriers	2	127	2	1	7
Cell envelope	2	194	5	10	10
Cellular process	1	102	18	14	17
Central intermediary metabolism	4	149	9	15	20
Energy metabolism	16	363	6	29	51
Fatty acid/Phospholipid metabolism	8	64	1	4	6
Nucleotide metabolism	11	120	2	14	20
Other categories	3	236	30	15	16
Regulatory functions	1	104	12	5	6
Replication	4	89	14	19	9
Transcription	1	47	5	7	5
Translation	0	150	2	61	16
Transport/binding protein	10	369	28	42	40
Total	65	2248	134	241	237
